# Physician experience with once-weekly somatrogon versus once-daily rhGH regimen in pediatric patients with growth hormone deficiency: a cross-sectional survey of physicians from the global phase 3 study

**DOI:** 10.3389/fendo.2023.1254424

**Published:** 2023-10-17

**Authors:** Roy Gomez, Roger Lamoureux, Diane M. Turner-Bowker, Jane Loftus, Mohamad Maghnie, Bradley S. Miller, Michel Polak, Andrew Yaworsky

**Affiliations:** ^1^ Global Medical Affairs, Pfizer Pte Limited, Singapore, Singapore; ^2^ Adelphi Values, Patient-Centered Outcomes, Boston, MA, United States; ^3^ Outcomes Research, Pfizer Ltd, Walton Oaks, Tadworth, United Kingdom; ^4^ Department of Pediatrics, IRCCS Istituto Giannina Gaslini, Genoa, Italy; ^5^ Department of Neuroscience, Rehabilitation, Ophthalmology, Genetics, Maternal and Child Health, University of Genoa, Genoa, Italy; ^6^ Division of Endocrinology, Department of Pediatrics, University of Minnesota Medical School, M Health Fairview Masonic Children’s Hospital, Minneapolis, MN, United States; ^7^ D’endocrinologie, Gynécologie et Diabétologie Pédiatriques, Hôpital Universitaire Necker Enfants Malades, Paris, France

**Keywords:** growth hormone deficiency, long-acting growth hormone, once-weekly injection regimen, pediatric endocrinology, somatrogon, somatropin, survey, treatment experience

## Abstract

**Introduction:**

The standard of care for pediatric growth hormone deficiency (pGHD) is once-daily recombinant human growth hormone (rhGH). Somatrogon, a long-acting rhGH, requires less frequent, once-weekly, dosing. We describe physicians’ preference for, experiences, and satisfaction with once-weekly somatrogon vs once-daily rhGH.

**Methods:**

English-speaking investigators from somatrogon’s global phase III study (NCT02968004) with prior experience using once-daily rhGH were included. Participants answered an online survey containing 14 closed- and open-ended items.

**Results:**

Twenty-four pediatric endocrinologists (41.7% men; 79.2% practiced at public/private hospitals) from 12 countries with 25.8 ± 12.0 years’ experience treating pGHD completed the survey. In terms of the time and effort required to explain device instructions, injection regimen, procedure for missed injection, and address patients’/caregivers’ concerns, a similar proportion of physicians chose once-weekly somatrogon and once-daily rhGH; 62.5% physicians indicated that once-daily rhGH required greater effort to monitor adherence. Overall, 75% preferred once-weekly somatrogon over once-daily rhGH, 79.2% considered once-weekly somatrogon to be more convenient and less burdensome, and 83.3% were likely to prescribe somatrogon in the future. Overall, 50% felt that once-weekly somatrogon was more beneficial to patients, while 50% chose “No difference”. Most physicians (62.5%) felt both regimens were equally likely to support positive long-term growth outcomes and reduce healthcare utilization. More physicians were “very satisfied” with once-weekly somatrogon (62.5%) than with once-daily rhGH (16.7%). Reduced injection frequency, patient and caregiver burden, increased convenience, and improved adherence were reasons for these choices.

**Conclusion:**

Physicians had a positive experience with, and perception of, treating pGHD with once-weekly somatrogon.

## Introduction

One of the most frequent reasons for patient referrals to pediatric endocrinologists is short stature (1). Among the many possible causes of short stature, pediatric growth hormone deficiency (pGHD), characterized by inadequate circulating levels of growth hormone (GH) and insulin-like growth factor-1, represents the most common pituitary hormone deficiency in children (2). Pediatric GHD can be isolated or occur along with deficiency of other pituitary hormones. Isolated GHD is a rare disorder with a prevalence of 1:4000 to 1:10,000 (3–5).

Pediatric GHD generally manifests as short stature and growth failure (2). Aside from short stature, children with GHD may have unfavorable alterations in metabolism and body composition, such as impaired lipid profile (6, 7); heightened insulin sensitivity, particularly when young (8); and increased visceral adiposity (9). Hypoglycemic episodes occur in approximately 5% of children with GHD, predominantly in infancy (10). Given that the main morbidity associated with pGHD is short stature, the primary goals of treatment are to accelerate height velocity to promote linear growth during childhood and achieve an adult height appropriate for the child’s genetic potential (11).

Early diagnosis and treatment of pGHD is highly effective for increasing childhood height and attaining final adult height (12, 13). Consequently, guidelines recommend starting pGHD treatment as soon as a pGHD diagnosis is made (14). The standard of care for treating pGHD over the past 30 years has been recombinant human growth hormone (rhGH) or somatropin (11), which has an amino acid sequence identical to that of the naturally occurring human growth hormone (hGH) (15). In order to optimize growth rates, subcutaneous injections of rhGH are recommended to be administered in the evening on a daily basis (14, 15). This daily rhGH regimen has been demonstrated to promote linear growth in pGHD and maintain a healthy body composition, normal blood glucose levels, and a favorable lipid profile (15). However, treatment outcomes for daily rhGH injections are influenced by treatment adherence rates, which vary widely in children and from childhood to adolescence (16). A US population-based retrospective study evaluated the adherence to daily rhGH treatment in children with GHD, aged ≥3 and <16 years, through 4 years of follow-up. Suboptimal adherence was specified as medication possession ratio <80%, and discontinuation was defined as the first occurrence of a gap of more than 60 days between rhGH prescription fills. Suboptimal adherence was found to increase over time (19.6% at year 1 and 35.9% at year 4). Over the 4-year period, 42.2% of the patients discontinued therapy, with a median time to discontinuation of 1.2 years. Discontinuation rates were higher for those aged 10 years and older, females, African-American and Hispanic patients, and patients who are obese (17).

Low treatment adherence is a significant cause of the suboptimal therapeutic response to daily rhGH therapy, and failure to adhere to the prescribed treatment schedule may interfere with the efficacy of pGHD therapy (16). Daily rhGH injections constitute a substantial treatment burden on patients and caregivers. This treatment burden may include the time and effort required to plan, prepare, and administer each dose; injection-related pain; psychological burden; and impact on life’s daily activities, and can lead to a decline in adherence over the years (18). Consequently, it appears that decreased injection frequency and a simpler treatment regimen have the potential to improve adherence and clinical outcomes (19). In this context, the recently developed long-acting growth hormone (LAGH) analogs are a promising treatment option as they decrease the frequency of required injections and are hypothesized to improve treatment adherence, leading to better treatment outcomes (20).

Somatrogon is a long-acting rhGH containing the amino acid sequence of hGH and three copies of the carboxy-terminal peptide (CTP) from human chorionic gonadotropin (21). The CTPs extend the half-life of the attached rhGH, allowing longer intervals between doses (21). Somatrogon is currently approved in the European Union, Canada, Australia, Japan, Taiwan, Switzerland, the United Kingdom, the United Arab Emirates, India, and Brazil as a once-weekly subcutaneous injection for children with GHD. A 12-month, open-label, multicenter, randomized phase III trial (NCT02968004) in GH-treatment-naïve prepubertal children with GHD compared the efficacy and safety of once-weekly somatrogon with once-daily somatropin (Genotropin^®^). The study demonstrated that once-weekly somatrogon was non-inferior to once-daily somatropin, and was well tolerated in patients with pGHD (22). After completing 12 months in the main study, patients had the option to enter the long-term, open-label extension of the study.

Considering that physicians’ perceptions and experiences of treatment are key drivers of the use of GH therapy in adults in clinical practice (23), it is imperative to understand physicians’ perspectives and experiences with once-weekly somatrogon and how they regard this regimen for the treatment of children. In keeping with this objective, we surveyed pediatric endocrinologists who had prior experience in treating children with GHD with once-daily rhGH and had treated children with GHD randomized to once-weekly somatrogon as part of the pivotal phase III trial.

The objectives of this study were to explore and describe: (1) physicians’ perspectives using once-weekly somatrogon to treat pediatric patients with GHD, and (2) physicians’ preference for, and satisfaction with, once-weekly somatrogon, compared with once-daily rhGH.

## Methods

This was a cross-sectional, observational study that quantitatively assessed physicians’ experience with once-weekly somatrogon and once-daily rhGH in pediatric patients with GHD, via an online survey.

English-speaking physicians who were clinical investigators in the phase III trial (NCT02968004) sponsored by OPKO Health were eligible for inclusion if they had treated at least two children with GHD with once-weekly somatrogon as part of the phase III trial, either during the randomization phase or the open-label extension phase of the study and had prior experience with using once-daily rhGH. Up to 50 eligible physicians were planned to be enrolled in the study.

### Survey development

The Healthcare Provider Growth Hormone Treatment Experience Survey was developed by a team comprising representatives from the study sponsor and experts in patient-centered outcomes research. The initial content of the survey was informed by examination of the results of a literature review (24) focused on the experiences of patients switching from a daily treatment to a less frequent treatment regimen. Based on the review findings, the preliminary survey content was drafted by the research team and consisted of 16 items. This content was subsequently revised based on feedback provided at an advisory board meeting attended by members of the research team and four pediatric endocrinologists experienced in treating patients with GHD. Following advisory board input, the number of items included in the survey were reduced to 14; minor wording changes were also made at this stage to improve clarity and ease of response.

The final survey is a 14-item questionnaire, designed for online administration, that assesses physicians’ experiences, preferences, and satisfaction with the use of once-weekly somatrogon compared with once-daily rhGH. Seven of these 14 items include open-ended, free-text fields in which physicians can describe why they chose their answer. The final survey questionnaire is provided in the online **Supplementary Material**.

Along with the survey, two additional study forms (physician-informed consent form [P-ICF] and clinician screener and demographic form [CS-DF]) were developed and programmed for electronic administration via a Health Insurance Portability and Accessibility Act (HIPAA)-compliant online platform – SurveyMonkey.

Pediatric endocrinologists from 55 sites in 19 countries who previously participated in the phase III study (NCT02968004) were contacted with information about the study; those physicians who had been involved in the development of the survey were excluded from participation. Interested pediatric endocrinologists were provided with a secure hyperlink containing the P-ICF and CS-DF. First, physicians were asked to read and complete the P-ICF, and those who consented to participate in the study (via the P-ICF) were directed to complete the CS-DF to confirm their eligibility for the survey. Once confirmed, they were granted access to answer the survey. To minimize missing data, each survey question had to be completed before the next question was made available. Physicians were able to move between questions, as needed, to review and revise previous responses. Physicians who completed the survey were compensated for their time spent, based on the fair market value rate for the physician’s country.

### Ethics

All study documents were approved by an independent ethical review board, Sterling IRB, with the IRB ID number 9045-RELamoureux. This study complied with all applicable laws, including laws on the implementation of organizational and technical measures to ensure protection of physicians’ personal data. Physicians’ names or other directly identifiable data were omitted from reports, publications, or other disclosures, except where required by applicable laws. Study data were presented in an aggregated and anonymized manner.

### Participants

#### Inclusion criteria

Physicians were eligible if they: (1) provided a signed and dated P-ICF, (2) had treated at least two children (aged ≥3 years and <11 years [for girls]/<12 years [for boys] on the date of P-ICF signature) diagnosed with GHD with once-weekly somatrogon at a clinical site in the phase III trial (NCT02968004), either during the randomization phase or the open-label extension phase, (3) had experience of treating patients with pGHD with once-daily rhGH, and (4) were fluent in English.

#### Exclusion criteria

Pediatric endocrinologists who participated in the development of the survey were excluded. A full list of exclusion criteria is provided in the online **Supplementary Material**.

### Survey questions

The 14-item Healthcare Provider Growth Hormone Treatment Experience Survey consists of four distinct parts. The first part includes five items that ask physicians to compare both injection regimens and rate, using a five-point Likert scale, the time and effort required to: (1) explain the device’s instructions for use (IFU), (2) explain the injection regimen, (3) explain what to do if an injection is missed, (4) address patients’ and caregivers’ concerns, and (5) monitor treatment adherence.

The second part of the survey comprises a set of three items that assess physicians’ preference for once-weekly somatrogon or once-daily rhGH in terms of general preference, convenience, and burden. The third part of the survey assesses physicians’ overall treatment preference for once-weekly somatrogon or once-daily rhGH using four items. The first item asks the physician to choose the injection regimen that the physician would be more likely to prescribe in the future. The remaining three items assess the physician’s perspective on which of the two injection regimens is more likely to be beneficial to patients, support positive long-term outcomes, and reduce healthcare utilization. For the second and third parts of this survey, physicians are asked to choose between the two injection regimens or select “No difference”. Each of the seven items in the second and third parts of the survey also includes an open-ended, free-text field in which physicians can describe the rationale for their selected response.

The last part of the survey contains two items that assess physicians’ satisfaction with each injection regimen using a five-point Likert scale, ranging from “Very satisfied” to “Very dissatisfied”. The complete survey questionnaire is provided in the online **Supplementary Material**.

### Statistical analyses

A targeted sample of up to 50 physicians was planned for recruitment to complete the survey. Categorical variables were summarized using frequencies and percentages, and continuous variables were summarized using descriptive statistics. Quantitative analysis of survey data was performed using SAS v9.4 software (SAS Institute, Cary, NC, United States). Data entered into free-text fields were coded using ATLAS.ti v9 software (ATLAS.ti, Berlin, Germany) in preparation for qualitative analysis (25–27).

## Results

Survey data were collected between June 25, 2021 and November 11, 2021.

### Participants

A total of 54 pediatric endocrinologists were invited to take part in the survey. Of these, 16 did not respond to the invitation; three were ineligible; one physician had retired, and therefore, did not participate; four declined to participate; and six physicians who were willing to participate and were provided with access to the survey, did not complete it. The percentage of physicians who responded to the survey invitation was 70.4%, and, among respondents who received the survey, 80.0% completed the survey.

Overall, 24 physicians (10 men, 14 women) from 12 countries (Bulgaria, Canada, Colombia, Greece, India, Israel, Mexico, Russia, South Korea, Spain, Ukraine, and the United States) completed the survey. Participating physicians had 25.8 (8.0–60.0) years of experience treating pGHD (**Table 1**), and the mean number of patients being treated at the time of the survey was 141. The majority of the physicians practiced at a public (41.7%) or private hospital (37.5%) (**Table 1**).

**Table 1:** Participating physicians’ characteristics (N=24).

Characteristic	Value
Number of years practicing endocrinology	
Mean (SD)	27.2 (9.7)
Median	28
Minimum, maximum	8.0, 50.0
Number of years treating pediatric patients with GHD	
Mean (SD)	25.8 (12.0)
Median	25
Minimum, maximum	8.0, 60.0
Type of practice setting^a^, n (%)	
Hospital (private)	9 (37.5)
Hospital (public)	10 (41.7)
Group clinic practice	5 (20.8)
Individual clinic	7 (29.2)
Other	3 (12.5)

a Participants could choose more than one practice setting.

GHD, growth hormone deficiency; SD, standard deviation.

### Physicians’ experience with using once-weekly somatrogon vs once-daily rhGH

**Figure 1:**
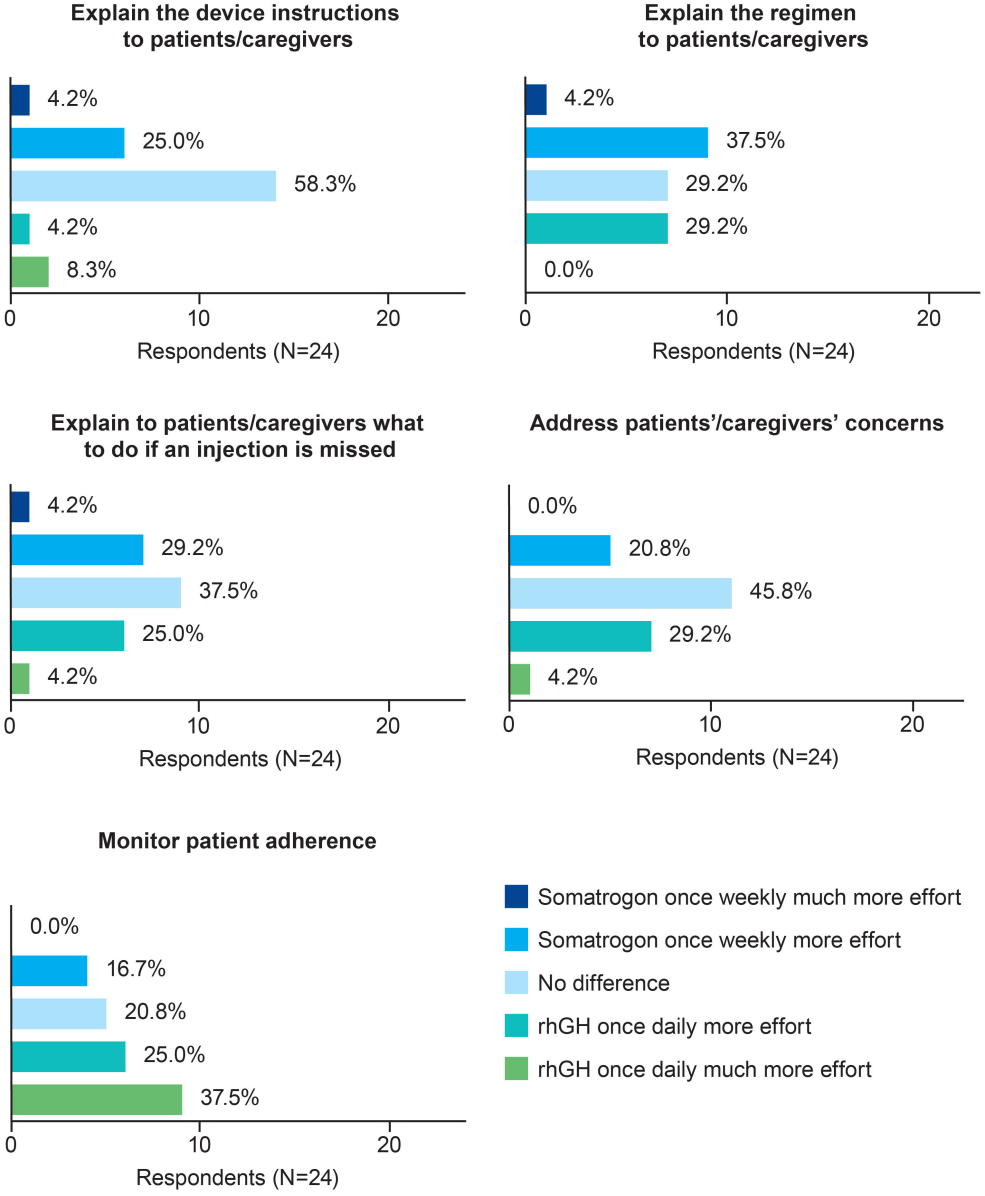
Physicians’ perceptions of the time and effort needed to prescribe once-weekly somatrogon vs once-daily rhGH and monitor adherence. rhGH, recombinant human growth hormone.

Most physicians (n=14; 58.3%) perceived no difference between the two regimens in terms of the effort needed to explain the injection device IFU to patients or caregivers (**Figure 1**). In terms of the effort required to explain the injection regimen, what to do if an injection is missed, and to address patients’ and caregivers’ questions, the proportion of physicians who chose once-weekly somatrogon was similar to the proportion who chose once-daily rhGH (**Figure 1**). Over half of the physicians (n=15; 62.5%) reported that daily rhGH injections required greater or much greater effort to monitor adherence compared with once-weekly somatrogon injections (**Figure 1**). Five physicians (20.8%) reported no difference between the two regimens in terms of the effort required to monitor adherence.

### Physicians’ preferences for once-weekly somatrogon vs once-daily rhGH

#### Preferred injection regimen

**Figure 2:**
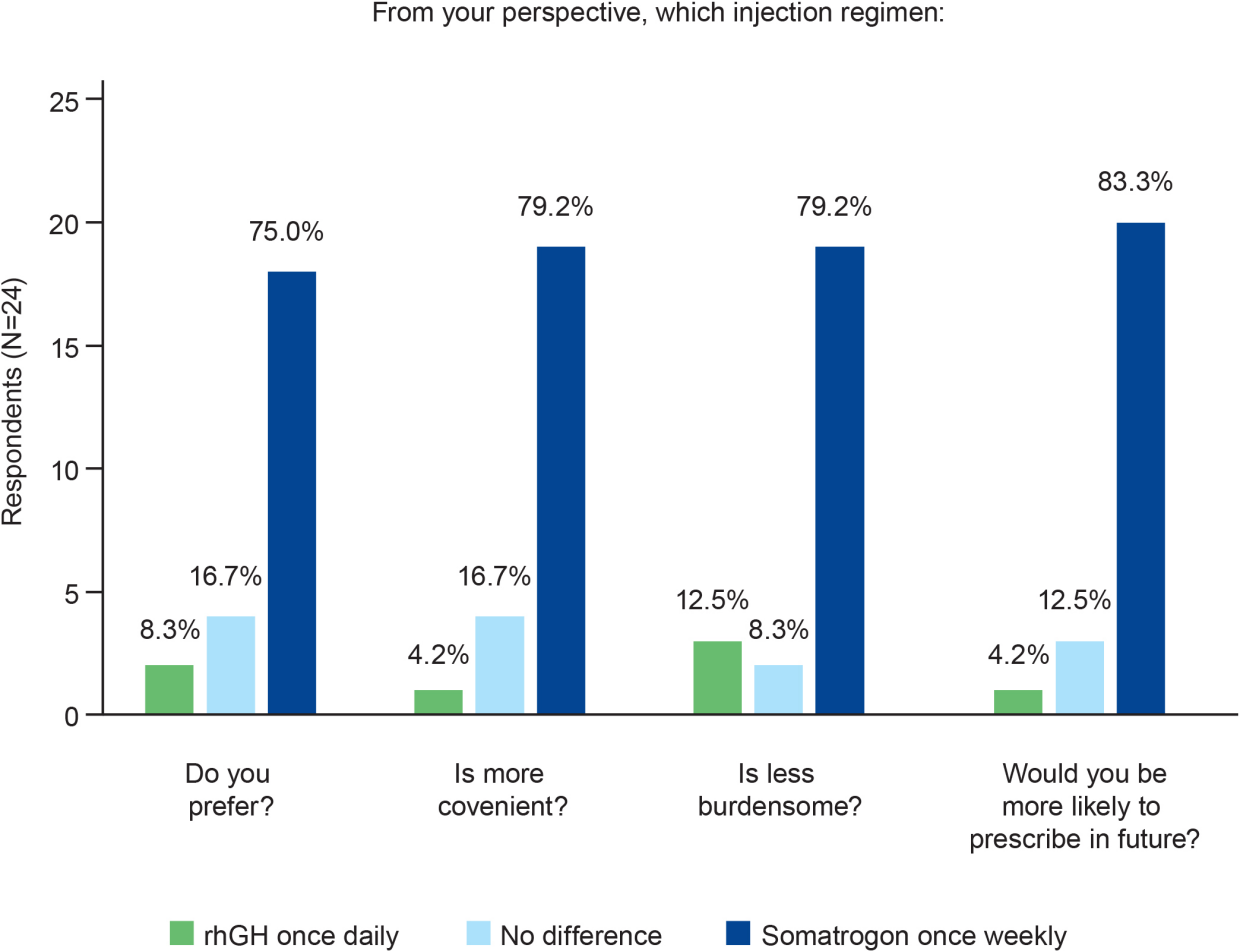
Physicians’ preferences regarding the use of once-weekly somatrogon and once-daily rhGH. rhGH, recombinant human growth hormone.

Three-quarters of the physicians (n=18; 75.0%; **Figure 2**) expressed a preference for once-weekly somatrogon over once-daily rhGH, citing the following reasons: once-weekly somatrogon would likely improve adherence (n=8, 33.3%); requires fewer injections (n=4, 16.7%); decreases caregiver burden (n=3, 12.5%); has better patient acceptance (n=3, 12.5%); and is more convenient (n=3, 12.5%). Four physicians (16.7%) chose “No difference” as the response. One of the physicians explained that this was because “Daily injections are easier for patients to remember. Weekly injections reduce pain”. Another physician explained that LAGH has the same efficacy and injection discomfort as daily GH, although they indicated that they had limited experience with LAGHs. Two physicians (8.3%) preferred the once-daily rhGH over once-weekly somatrogon. One of them reported that daily injections were less painful and require a lower total dose for a similar effect, and that daily dosing produces a GH profile that is similar to the physiologic secretion pattern.

#### Convenience and burden associated with injection regimen

Most physicians (n=19; 79.2%) considered once-weekly somatrogon to be more convenient as well as less burdensome than once-daily rhGH (**Figure 2**). The most common reasons cited by physicians for this choice were that somatrogon requires fewer injections, improves adherence, decreases caregiver burden, decreases daily burden, and requires less time to administer.

#### Injection regimen more likely to be prescribed

The majority (n=20; 83.3%) of physicians responded that they would be more likely to prescribe somatrogon in the future compared with once-daily rhGH (**Figure 2**). Improved adherence (n=7), the need for fewer injections (n=6), and greater convenience (n=3) were some of the most frequently cited reasons that physicians would be more likely to prescribe somatrogon. Three physicians (12.5%) indicated that there was no difference in the likelihood of them prescribing either regimen. One physician mentioned that they would explain both treatments to the parents and allow them to decide. Another physician mentioned that for GH-naïve patients, the physician would offer both treatments. The third physician mentioned that the choice would depend on the patient’s age and the hospital’s budget, considering they have a public health system.

### Physicians’ overall treatment preference

#### Injection regimen felt to be more beneficial to patients

**Figure 3:**
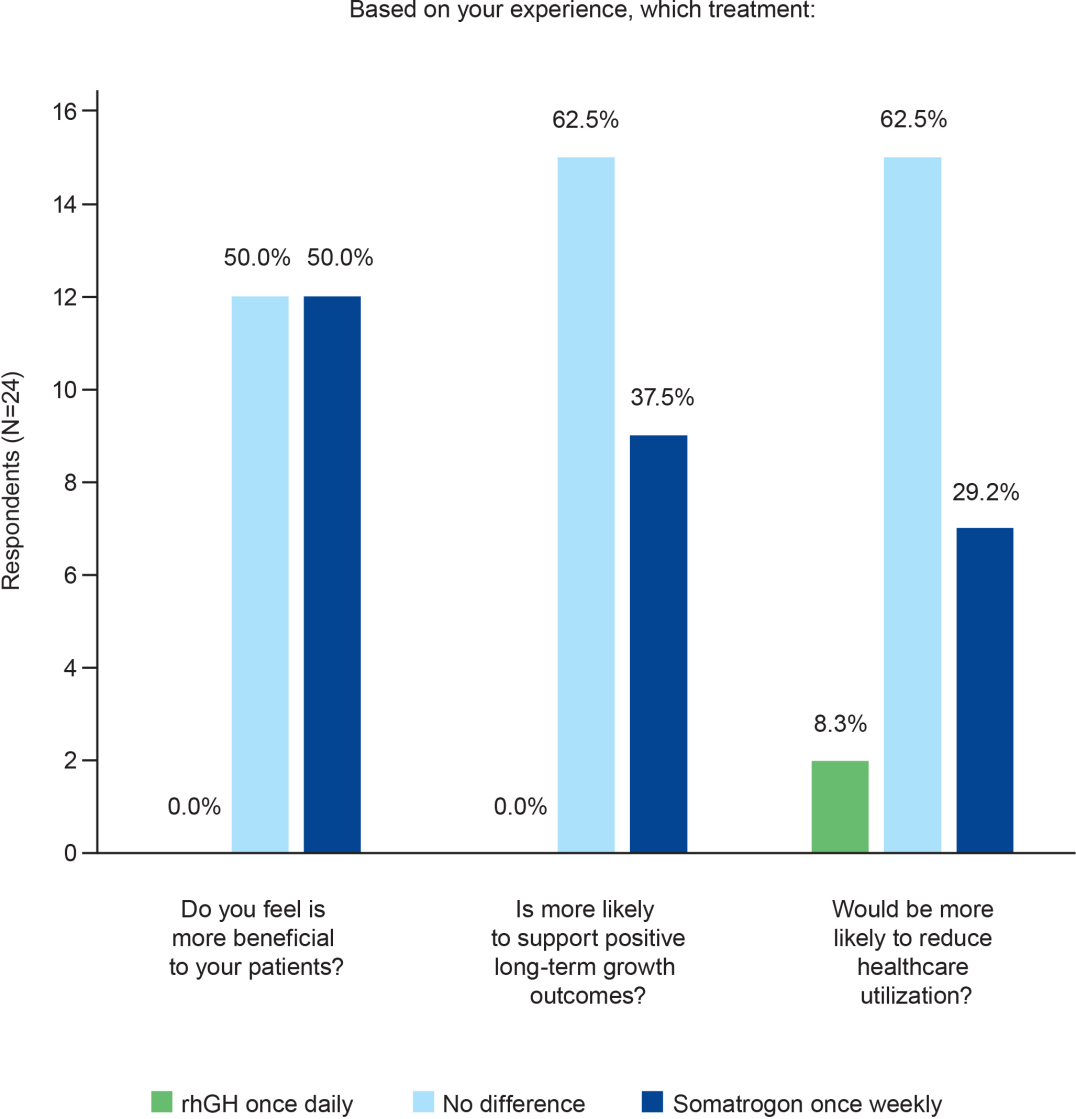
Physicians’ perceptions of treatment benefit. rhGH, recombinant human growth hormone.

Half of the physicians (n=12) felt that once-weekly somatrogon was more beneficial to their patients (**Figure 3**). This was because the need for fewer injections would translate to better treatment acceptability, improved patient compliance, and decreased drop-out rate, and that all these factors would eventually lead to enhanced quality of life and improved growth. The other half of the physicians surveyed felt that there was no difference in benefit between the two treatment regimens (**Figure 3**). Eight physicians (33.3%) perceived the efficacy to be similar for the daily and weekly regimens, and one physician indicated that both regimens were equally safe. Two physicians mentioned that they would need more data for somatrogon before making a decision.

#### Injection regimen perceived as more likely to support positive long-term outcomes

Most physicians (n=15; 62.5%) felt that the two regimens were equally likely to support positive long-term growth outcomes (**Figure 3**). However, over one-third (n=9; 37.5%) of the physicians considered somatrogon to be more likely to support these outcomes.

#### Injection regimen thought to reduce healthcare utilization

In terms of reducing healthcare utilization, most physicians (n=15; 62.5%) thought that there would likely be no difference between the two regimens. Seven physicians (29.2%) felt that once-weekly somatrogon was more likely to reduce healthcare utilization (**Figure 3**).

### Physicians’ satisfaction with each regimen

**Figure 4:**
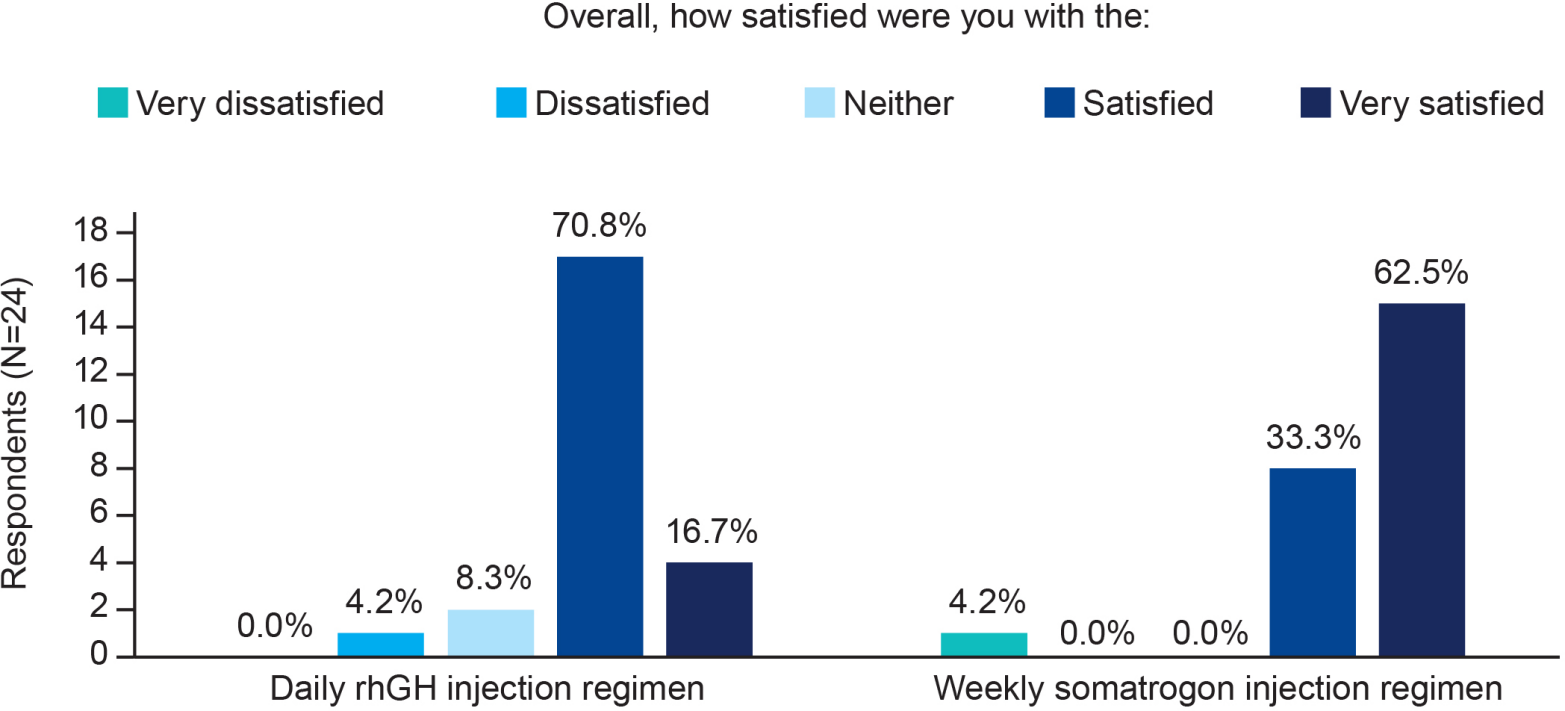
Physicians’ satisfaction with the use of once-weekly somatrogon and once-daily rhGH. rhGH, recombinant human growth hormone.

A higher proportion of physicians were “very satisfied” with once-weekly somatrogon as compared with once-daily rhGH (62.5% [n=15] vs 16.7% [n=4], respectively; **Figure 4**). All physicians except for one (n=23; 95.8%) were either “satisfied” or “very satisfied” with once-weekly somatrogon, whereas 21 physicians (87.5%) were either “satisfied” or “very satisfied” with the once-daily rhGH (**Figure 4**).

## Discussion

To our knowledge, this is the first global survey comparing physicians’ experiences and perspectives of the once-weekly somatrogon regimen with the current standard of care – once-daily rhGH – in children with GHD. The findings of this study indicate that the once-weekly somatrogon regimen was generally preferred and was easier for physicians to monitor compared with the once-daily rhGH regimen. Overall, the majority of physicians preferred once-weekly somatrogon vs once-daily rhGH (75.0% vs 8.3%, respectively) and reported the once-weekly somatrogon regimen to be more convenient (79.2% vs 4.2%, respectively) and less burdensome (79.2% vs 12.5%, respectively). More than half of the physicians (62.5%) reported that less effort is required to monitor adherence in patients with once-weekly somatrogon compared with once-daily rhGH. Half of the physicians felt that once-weekly somatrogon was more beneficial to their patients, and most of the physicians (62.5%) perceived no difference between the regimens in terms of being more likely to support positive long-term growth outcomes or reduce healthcare utilization. Overall, a very large proportion of physicians were “very satisfied”/“satisfied” with both once-weekly somatrogon (95.8%) and once-daily rhGH (87.5%).

Willingness to prescribe once-weekly somatrogon in the future was reported by the majority of physicians compared with once-daily rhGH (83.3% vs 4.2%, respectively). This finding aligns with the previously highlighted feedback around convenience and burden, but the results of our study also highlight other aspects of the once-weekly somatrogon regimen that support this perspective. Based on the open-ended free-text responses, physicians felt that the use of once-weekly somatrogon may reduce the burden of chronic daily injections for children. Physicians’ positive responses to once-weekly somatrogon were also driven by reasons related to decreased caregiver burden, better patient acceptance, a higher level of convenience, and improved adherence, indicating that these factors, in addition to efficacy, are important determinants of physicians’ perceptions of pGHD treatment. This study highlights the relevance of physician and patient factors in the preference for, and selection of, pediatric GH therapy as well as physicians’ beliefs that once-weekly somatrogon has the potential to address the long-standing unmet need for increased treatment adherence, and hence improved treatment outcomes, with GH therapy.

To date, few, if any studies have assessed long-term patient adherence and treatment outcomes with a LAGH analog, likely because LAGH analogs in pGHD, such as lonapegsomatropin and somatrogon, have only recently received approval (28, 29). Despite limited data, there seems to be general agreement that patient adherence is an important element that physicians consider when selecting a GH product (23, 30). Pediatric GHD therapy is a long-term undertaking, and adhering to daily rhGH injections is demanding for the child and family, with adherence shown to decline over time. The use of once-weekly somatrogon to treat children with GHD may increase adherence and ease the burden of persistent daily injections (20). Our results show that physicians believed that the once-weekly somatrogon regimen was likely to improve adherence. Additionally, physicians who felt that somatrogon would be more beneficial to their patients cited increased adherence and reduced drop-out rates as significant reasons for this expectation.

In order to optimize pGHD therapy, pediatric endocrinologists use their clinical judgment and previous experience with GH treatment, in addition to being receptive and responsive to patients’ and caregivers’ perceptions of and concerns with the therapy. According to the open-ended responses to the survey, other reasons physicians favored somatrogon included patient acceptance of the treatment; reduced patient and caregiver burden, including decreased injection frequency; increased freedom that results from a simplified treatment regimen, such as the flexibility of a sleepover, traveling without the restrictions of a daily injection, patient care within split households, and the freedom to administer somatrogon on a weekend, with no interference on school days. These findings are consistent with previous studies demonstrating that GH product selection decisions were based on physicians’ perceptions of patients’ treatment acceptance (23, 30, 31). In a study evaluating reasons for GH selection in adults following pituitary surgery, preferences were found to be based on patient compliance, personal preferences, perceptions of what would be most acceptable to their patients, availability of GH injections in their health centers or hospitals, and adverse effects of therapy (23). Another study in primary and secondary care centers in England found that the key driver for specific GH preparation selection was the ease of use and number of steps in dose preparation, while the price of GH products was not the main factor influencing prescribing (31).

From the patient perspective, a less frequent injection schedule has previously been indicated as a preferred factor among adult and pediatric patients with GHD in a discrete choice experiment study in the United States (19). A recent study (32) that investigated the patients’ and caregivers’ perceptions of treatment burden associated with once-weekly somatrogon vs once-daily rhGH, reported that once-weekly somatrogon had a significantly lower treatment burden, based on mean overall life interference total scores. In the same study, once-weekly somatrogon was associated with a more favorable treatment experience overall compared with once-daily rhGH; for example, a greater number of patients preferred it, finding it to be more convenient and easier to follow, and a higher number of patients indicated their intention to comply with the once-weekly treatment.

Based on the studies described above, it appears that ease of use of the treatment and patient comfort are key considerations when physicians make prescribing decisions. In our survey, most physicians indicated that there was no difference between once-weekly somatrogon and once-daily rhGH in terms of the time and effort required to explain the injection device IFU and the treatment regimen to patients and caregivers. However, these findings should be viewed in context of the limited use of somatrogon to date. It is possible that differences between the two treatment regimens may be more discernible with continued use of, and familiarity with, once-weekly somatrogon. A few physicians expressed their concern related to the non-physiologic (elevated and non-pulsatile) GH exposure with somatrogon relative to daily rhGH. The physiologic secretion pattern of GH is episodic and pulsatile, exhibiting a diurnal rhythm, with approximately two-thirds of the total daily GH secretion occurring in major pulses during slow-wave sleep in the night (33). In this context, it is worth noting that none of the available daily rhGH preparations used to treat pGHD truly mimic the physiologic secretion pattern as they are not administered in a pulsatile manner, but are given as a single daily dose (34).

One of the strengths of our study is its international scope, with information collected about the experiences of physicians operating in different settings from over 10 different countries. The response and completion rates were also high (70.3% and 80.0%, respectively). Another strength is that physicians’ responses were based on their direct experience, as the study was limited to physicians with significant prior experience of treating children with GHD with once-daily rhGH and with once-weekly somatrogon in the phase III study. Given the cross-sectional nature of the study, another strength is the ability to quickly obtain and compile information about the study physicians’ experiences with once-weekly somatrogon.

Our study has several limitations. First, our study relies on physicians’ recall and opinion. There was variability in the length of time between a physicians’ last treatment experience and their completion of the survey. Physicians who treated patients at a more distant time point may have had less accurate recall compared with those with more recent experiences. Second, the study enrolled physicians who treated children with GHD in the pivotal phase III study, restricting their experience with once-weekly somatrogon to a clinical trial setting in a few patients as compared with the significant experience they had gained with once-daily rhGH, the standard of care for many years. It is worth noting that at the time this survey was conducted, somatrogon (NGENLA™) had not been marketed, and consequently, it remains to be assessed whether real-world experience with once-weekly somatrogon differs from that of a clinical trial setting. Third, since completion of the survey was voluntary, these study results may not reflect the experience of all the eligible physicians in the phase III trial. Fourth, due to the limited sample size and the small number of participating physicians in each country, statistical comparisons were not performed, and country trends were not analyzed or presented. Fifth, since the physicians participating in the survey were investigators in the somatrogon clinical trial, they may have a bias toward the potential benefits of LAGH therapy.

In conclusion, the results of our survey study demonstrate that participating physicians had a favorable perception of, and positive experience with, treating children with GHD with once-weekly somatrogon. Compared with once-daily rhGH, physicians found once-weekly somatrogon to be more convenient and less burdensome for patients. Most physicians had a clear preference for once-weekly somatrogon and were likely to prescribe somatrogon if it were available to them. Almost all physicians were “very satisfied”/“satisfied” with the once-weekly somatrogon regimen. Results from this study provide initial insights into physicians’ experience and perspectives of once-weekly somatrogon treatment. Future clinical practice studies are needed to characterize the real-world, long-term treatment experience with the once-weekly somatrogon injection regimen.

## Data availability statement

The original contributions presented in the study are included in the article/**Supplementary Material**. Further inquiries can be directed to the corresponding author. Upon request, and subject to review, Pfizer will provide the data that support the findings of this study. Subject to certain criteria, conditions and exceptions, Pfizer may also provide access to the related individual de-identified participant data. See https://www.pfizer.com/science/clinical-trials/trial-data-and-results for more information.

## Ethics statement

All study documents were approved by an independent ethical review board, Sterling IRB, with the IRB ID number 9045-RELamoureux. All physicians who completed the survey signed a physician informed consent form. This study complied with all applicable laws, including laws on the implementation of organizational and technical measures to ensure protection of physicians’ personal data.

## Author contributions

RG: Conceptualization, Methodology, Formal analysis, Investigation, Writing – review & editing, Supervision. RL: Methodology, Formal analysis, Investigation, Writing – review & editing. DT–B: Conceptualization, Methodology, Formal analysis, Writing – review & editing. JL: Conceptualization, Methodology, Formal analysis, Writing – review & editing. MM: Formal analysis, Writing – review & editing. BSM: Formal analysis, Writing – review & editing. MP: Formal analysis, Writing – review & editing. AY: Conceptualization, Methodology, Formal analysis, Investigation, Writing – review & editing.

## References

[1] Chiniara L Perry RJ Van Vliet G Huot C Deal C. Quality of referral of short children to the paediatric endocrinologist and impact of a fax communication system.Paediatr. Child Health (2013) 18:533–7. doi: 10.1093/pch/18.10.533 PMC3907349 24497780

[2] Calabria A. Growth hormone deficiency in children (pituitary dwarfism) (2022). Available at: https://www.msdmanuals.com/en-in/professional/pediatrics/endocrine-disorders-in-children/growth-hormone-deficiency-in-children (Accessed October 10, 2022).

[3] Stanley T. Diagnosis of growth hormone deficiency in childhood. Curr Opin Endocrinol Diabetes Obes (2012) 19:47–52. doi: 10.1097/MED.0b013e32834ec952 22157400 PMC3279941

[4] Lindsay R Feldkamp M Harris D Robertson J Rallison M. Utah Growth Study: growth standards and the prevalence of growth hormone deficiency. J Pediatr (1994) 125:29–35. doi: 10.1016/s0022-3476(94)70117-2 8021781

[5] Bao XL Shi YF Du YC Liu R Deng JY Gao SM. Prevalence of growth hormone deficiency of children in Beijing. Chin Med J (Engl) (1992) 105:401–5. 1499371

[6] Metwalley KA Farghaly HS Abd El-Hafeez HA. Evaluation of left ventricular mass and function, lipid profile, and insulin resistance in Egyptian children with growth hormone deficiency: a single-center prospective case-control study. Indian J Endocrinol Metab (2013) 17:876–82. doi: 10.4103/2230-8210.117234 PMC3784872 24083170

[7] Capalbo D Mattace Raso G Esposito A Di Mase R Barbieri F Meli R. Cluster of cardiometabolic risk factors in children with GH deficiency: a prospective, case-control study. Clin Endocrinol (Oxf) (2014) 80:856–62. doi: 10.1111/cen.12393 24372071

[8] Husbands S Ong KK Gilbert J Wass JA Dunger DB. Increased insulin sensitivity in young, growth hormone deficient children. Clin Endocrinol (Oxf) (2001) 55:87–92. doi: 10.1046/j.1365-2265.2001.01298.x 11453956

[9] De Leonibus C De Marco S Stevens A Clayton P Chiarelli F Mohn A. Growth hormone deficiency in prepubertal children: predictive markers of cardiovascular disease. Horm Res Paediatr (2016) 85:363–71. doi: 10.1159/000444143 26960169

[10] Bamba V. Pediatric growth hormone deficiency (2018). Available at: https://emedicine.medscape.com/article/923688-overview (Accessed July 7, 2022).

[11] Grimberg A DiVall SA Polychronakos C Allen DB Cohen LE Quintos JB. Guidelines for growth hormone and insulin-like growth factor-I treatment in children and adolescents: growth hormone deficiency, idiopathic short stature, and primary insulin-like growth factor-I deficiency. Horm Res Paediatr (2016) 86:361–97. doi: 10.1159/000452150 27884013

[12] Pozzobon G Partenope C Mora S Garbetta G Weber G Barera G. Growth hormone therapy in children: predictive factors and short-term and long-term response criteria. Endocrine (2019) 66:614–21. doi: 10.1007/s12020-019-02057-x 31423546

[13] Boguszewski MCS. Growth hormone deficiency and replacement in children. Rev Endocr Metab Disord (2021) 22:101–8. doi: 10.1007/s11154-020-09604-2 33029711

[14] Growth Hormone Research Society. Consensus guidelines for the diagnosis and treatment of growth hormone (GH) deficiency in childhood and adolescence: summary statement of the GH Research Society. GH Research Society. J Clin Endocrinol Metab (2000) 85:3990–3. doi: 10.1210/jcem.85.11.6984 11095419

[15] Reh CS Geffner ME. Somatotropin in the treatment of growth hormone deficiency and Turner syndrome in pediatric patients: a review. Clin Pharmacol (2010) 2:111–22. doi: 10.2147/cpaa.S6525 PMC3262362 22291494

[16] Fisher BG Acerini CL. Understanding the growth hormone therapy adherence paradigm: a systematic review. Horm Res Paediatr (2013) 79:189–96. doi: 10.1159/000350251 23635797

[17] Loftus J Chen Y Alvir JMJ Chi L Dasgupta S Gupta A. Suboptimal adherence to daily growth hormone in a US real-world study: an unmet need in the treatment of pediatric growth hormone deficiency.Curr. Med Res Opin (2021) 37:2141–50. doi: 10.1080/03007995.2021.1982682 34569388

[18] Pampanini V Deodati A Inzaghi E Cianfarani S. Long-acting growth hormone preparations and their use in children with growth hormone deficiency. Horm Res Paediatr (2022). doi: 10.1159/000523791 35220308

[19] McNamara M Turner-Bowker DM Westhead H Yaworsky A Palladino A Gross H. Factors driving patient preferences for growth hormone deficiency (GHD) injection regimen and injection device features: a discrete choice experiment. Patient Prefer Adherence (2020) 14:781–93. doi: 10.2147/ppa.S239196 PMC7198440 32431492

[20] Yuen KCJ Miller BS Boguszewski CL Hoffman AR. Usefulness and potential pitfalls of long-acting growth hormone analogs. Front Endocrinol (Lausanne) (2021) 12:637209. doi: 10.3389/fendo.2021.637209 33716988 PMC7943875

[21] Hershkovitz O Bar-Ilan A Guy R Felikman Y Moschcovich L Hwa V. *In vitro* and *in vivo* characterization of MOD-4023, a long-acting carboxy-terminal peptide (CTP)-modified human growth hormone. Mol Pharm (2016) 13:631–9. doi: 10.1021/acs.molpharmaceut.5b00868 26713839

[22] Deal CL Steelman J Vlachopapadopoulou E Stawerska R Silverman LA Phillip M. Efficacy and safety of weekly somatrogon vs daily somatropin in children with growth hormone deficiency: a phase 3 study. J Clin Endocrinol Metab (2022) 107:e2717–2728. doi: 10.1210/clinem/dgac220 PMC9202717 35405011

[23] Ekhzaimy A Beshyah SA Al Dahmani KM AlMalki MH. Physician' attitudes to growth hormone replacement therapy in adults following pituitary surgery: results of an online survey. Avicenna J Med (2020) 10:215–22. doi: 10.4103/ajm.ajm_46_20 PMC7791281 33437693

[24] Loftus J Yaworsky A Roland CL Turner-Bowker D McLafferty M Su S. Systematic review of patient experience with a less frequent injection schedule for growth hormone deficiency. J Manag Care Spec Pharm (2021) 27:S122.

[25] Friese S. ATLAS.ti 9 Windows - user manual (2021). Available at: https://doc.atlasti.com/ManualWin.v9/ATLAS.ti_ManualWin.v9.pdf (Accessed December 8, 2022).

[26] Weitzman EA Miles MB. Computer Programs For Qualitative Data Analysis: A Software Sourcebook. Thousand Oaks, California: Sage Publications, Inc (1995).

[27] Willis GB. Cognitive Interviewing: A Tool For Improving Questionnaire Design. Thousand Oaks, California: Sage Publications, Inc (2005).

[28] Lamb YN. Lonapegsomatropin: pediatric first approval. Paediatr Drugs (2022) 24:83–90. doi: 10.1007/s40272-021-00478-8 34709591

[29] Lamb YN. Somatrogon: first approval. Drugs (2022) 82:227–34. doi: 10.1007/s40265-021-01663-2 35041176

[30] Nelson WW Frear RS. Physician attitudes toward human growth hormone products. Am J Health Syst Pharm (1999) 56:51–6. doi: 10.1093/ajhp/56.1.51 10048879

[31] Chapman SR Fitzpatrick RW Aladul MI. What drives the prescribing of growth hormone preparations in England? Prices versus patient preferences. BMJ Open (2017) 7:e013730. doi: 10.1136/bmjopen-2016-013730 PMC5541334 28400458

[32] Maniatis AK Carakushansky M Galcheva S Prakasam G Fox LA Dankovcikova A. Treatment burden of weekly somatrogon vs daily somatropin in children with growth hormone deficiency: a randomized study. J Endocr Soc (2022) 6:bvac117. doi: 10.1210/jendso/bvac117 36101713 PMC9463876

[33] Kaiser U Ho K. Pituitary physiology and diagnostic evaluation. In: Melmed S Polonsky K Larsen P Kronenberg H, editors. Williams Textbook Of Endocrinology. Philadelphia, Pennsylvania: Elsevier (2016). p. 176–231. doi: 10.1016/B978-0-323-29738-7.00008-3

[34] Yuen KCJ Miller BS Biller BMK. The current state of long-acting growth hormone preparations for growth hormone therapy. Curr Opin Endocrinol Diabetes Obes (2018) 25:267–73. doi: 10.1097/med.0000000000000416 29746309

