# Supplementary material for: Physician experience with once-weekly somatrogon versus once-daily rhGH regimen in pediatric patients with growth hormone deficiency: a cross-sectional survey of physicians from the global phase 3 study
Source: Front Endocrinol (Lausanne). 2023 Oct 17;14:1254424. doi: 10.3389/fendo.2023.1254424 (PMC10634585; doi:10.3389/fendo.2023.1254424)
Supplement: Supplementary file 1 [file DataSheet_1.pdf]

Supplementary Material

Full list of exclusion criteria

The exclusion criteria for the Healthcare Provider Growth Hormone Treatment Experience Survey evaluating physicians' experience with once-weekly somatrogon vs once-daily recombinant human growth hormone regimen in pediatric patients with growth hormone deficiency were:

- Physicians who participated in the development of the Healthcare Provider Growth Hormone Treatment Experience Survey.
- US-based physicians who were licensed in the state of Vermont or Maine (prohibited from compensation for participation).
- US-based physicians with primary practice within the Veterans Affairs or government setting (prohibited from receiving compensation).

Final survey questionnaire
Healthcare Provider Growth Hormone Treatment Experience Survey
Screen 1

Healthcare Provider Growth Hormone Treatment Experience Survey

Instructions

This survey includes questions that ask about your experience treating patients with growth hormone injections as part of the somatrogon clinical trial. As you answer each question, please consider **your experiences treating patients** with the once-daily injection regimen, and the somatrogon once-weekly injection regimen during the course of the trial.

For the following questions, consider **the amount of effort (time and energy)** required by you and your clinic staff to conduct the following study activities with patients and caregivers for daily injection treatment regimen compared to somatrogon weekly treatment regimen.

Note: The asterisk (*) indicates that this question must be completed before moving on to the next screen.

***1. How much effort (time and energy)** did it take for you and your clinic staff to…

	Much more effort for daily injection regimen	More effort for daily injection regimen	No difference	More effort for somatrogon weekly injection regimen	Much more effort for somatrogon weekly injection regimen
1a. Explain the injection device instructions for use (IFU) to patients and/or caregivers	○	○	○	○	○
1b. Explain the injection regimen to patients and/or caregivers (e.g., when and how frequently the dose should be taken, how to manage missed doses)	○	○	○	○	○
1c. Explain to patients and/or caregivers what to do if an injection is missed	○	○	○	○	○
1d. Monitoring patient adherence to growth hormone treatment	○	○	○	○	○
1e. Address patient and/or caregiver questions or concerns	○	○	○	○	○

Next

Screen 2

Please answer each of the following questions **based on your experience treating patients** during the somatrogon trial. Please think about the **treatment regimen** when selecting your answer.

Note: The asterisk (*) indicates that this question must be completed before moving on to the next screen.

***2. From your perspective**

	Daily injection regimen	No difference	Somatrogon weekly injection regimen
Which injection regimen do you prefer?	○	○	○
Why? (Please describe)			

Previous Next

Screen 3

Please answer each of the following questions **based on your experience treating patients** during the somatrogon trial. Please think about the **treatment regimen** when selecting your answer.

Note: The asterisk (*) indicates that this question must be completed before moving on to the next screen.

***3. From your perspective**

	Daily injection regimen	No difference	Somatrogon weekly injection regimen
Which injection regimen is more convenient?	◌	◌	◌
Why? (Please describe)			

Previous Next

Screen 4

Please answer each of the following questions **based on your experience treating patients** during the somatrogon trial. Please think about the **treatment regimen** when selecting your answer.

Note: The asterisk (*) indicates that this question must be completed before moving on to the next screen.

***4. From your perspective**

	Daily injection regimen	No difference	Somatrogon weekly injection regimen
Which injection regimen is less burdensome?	◌	◌	◌
Why? (Please describe)			

Previous Next

Screen 5

Please answer each of the following questions **based on your experience treating patients** during the somatrogon trial. Please think about the **overall treatment experience** when selecting an answer.

Note: The asterisk (*) indicates that this question must be completed before moving on to the next screen.

***5. Based on your experience…**

	Daily injection regimen	No difference	Somatrogon weekly injection regimen
Which treatment would you be more likely to prescribe for your patients in the future?	○	○	○
Why? (Please describe)			

Previous   Next

Screen 6

Please answer each of the following questions **based on your experience treating patients** during the somatrogon trial. Please think about the **overall treatment experience** when selecting an answer.

Note: The asterisk (*) indicates that this question must be completed before moving on to the next screen.

***6. Based on your experience…**

	Daily injection regimen	No difference	Somatrogon weekly injection regimen
Which treatment do you feel is more beneficial to your patients?	○	○	○
Why? (Please describe)			

Previous Next

Screen 7

Please answer each of the following questions **based on your experience treating patients** during the somatrogon trial. Please think about the **overall treatment experience** when selecting an answer.

Note: The asterisk (*) indicates that this question must be completed before moving on to the next screen.

***7. Based on your experience…**

	Daily injection regimen	No difference	Somatrogon weekly injection regimen
Which treatment is more likely to support positive long-term growth outcomes?	◌	◌	◌
Why? (Please describe)			

Previous Next

Screen 8

Please answer each of the following questions **based on your experience treating patients** during the somatrogon trial. Please think about the **overall treatment experience** when selecting an answer.

Note: The asterisk (*) indicates that this question must be completed before moving on to the next screen.

***8. Based on your experience…**

	Daily injection regimen	No difference	Somatrogon weekly injection regimen
Which treatment would be more likely to reduce healthcare utilization?	◌	◌	◌
Why? (Please describe)			

Previous Next

Screen 9

Please answer each of the following questions **based on your experience treating patients** during the somatrogon trial.

Please select only one response for each question.
Note: The asterisk (*) indicates that this question must be completed before moving on to the next screen.

***9. Overall, how satisfied were you with the…**

	Very satisfied	Satisfied	Neither satisfied nor dissatisfied	Dissatisfied	Very dissatisfied
9a. Daily injection regimen?	○	○	○	○	○
9b. Somatrogon weekly injection regimen?	○	○	○	○	○

Previous
